# Synergistic Effect of 5‑Fluorouracil and Amphotericin B in Murine Paracoccidioidomycosis: Immune Modulation and Enhanced Fungal Clearance

**DOI:** 10.1021/acsinfecdis.5c00944

**Published:** 2025-12-31

**Authors:** Filipe N. Franco, Ana Claudia S. dos Santos, Bianca V. dos Santos, Nycolas W. Preite, Coral Molist-Homs, Luiz Fernando F. de Oliveira, Bruno M. Borges, Flavio V. Loures

**Affiliations:** † Institute of Science and Technology, Federal University of São Paulo, São José dos Campos, CEP 12231-280, Brazil; ‡ Department of Biomedical Sciences and Pathobiology, Virginia Tech University, Blacksburg, Virginia 24060, United States

**Keywords:** P. brasiliensis, 5-fluorouracil, amphotericin, immunology, infectious diseases

## Abstract

Paracoccidioidomycosis (PCM) is a systemic fungal infection that primarily affects the lungs. Previous studies have shown that 5-fluorouracil (5-FU), a chemotherapeutic agent, reduces pulmonary myeloid-derived suppressor cells (MDSCs), thereby stimulating immune responses in PCM. This study aimed to evaluate the efficacy of the combined 5-FU and AmB therapy in a murine model of PCM. C57BL/6 mice were infected with *Paracoccidioides brasiliensis* and treated with AmB and/or 5-FU. We found that the 5-FU and AmB combination therapy led to improved disease control, as evidenced by reduced fungal burden, decreased tissue damage, and an increased survival rate. Moreover, the combined treatment was associated with decreased lymphocyte and neutrophil counts, along with an increased number of macrophages in pulmonary tissue, suggesting a controlled infectious process without hyperinflammatory reactions. These findings support the potential of combining 5-FU with conventional antifungal therapy as a promising strategy for enhancing PCM treatment outcomes.

## Graphical abstract

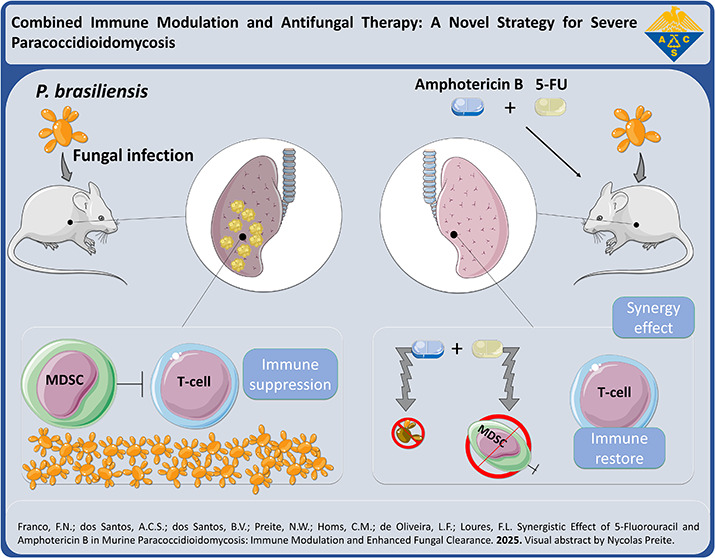

Paracoccidioidomycosis is a chronic systemic fungal infection caused by *Paracoccidioides* species, mainly *P. brasiliensis* and *P. lutzii*. It is characterized by polymorphic lesions and can affect several organs, including the skin, lymph nodes, lungs, and the oral, nasal, and gastrointestinal mucous membranes, adrenal glands, and central nervous system.^1^ Latin America stands out in the global scenario of cases, with Brazil having the highest number of PCM cases (80%), followed by Colombia, Venezuela, Ecuador, and Argentina.^2^ Furthermore, the disease is also reported in the extreme north of Mexico.^3^

Current treatment is based on the antifungal agents amphotericin B, fluconazole, and itraconazole. Amphotericin B (AmB) is primarily used for the treatment of acute/severe infections, while itraconazole is the first-line choice for mild to moderate infections. However, since current guidelines are primarily based on noncomparative studies and expert opinion and given the limited number of studies directly comparing itraconazole and fluconazole, there is a clear need for more robust clinical trials. A significant challenge in the treatment of PCM is the prolonged use of antifungals, which often results in poor patient adherence. Moreover, long-term treatment can lead to sequelae due to chronic inflammatory processes and fibrosis, which can impair the functions of affected organs such as the lungs. Furthermore, persistent stimulation of fungal antigen and activation of the immune system (IS) can lead to fibrosis through excessive deposition of extracellular matrix components and alterations in tissue healing, despite treatment.^4,5^

Numerous studies have demonstrated the involvement of myeloid-derived suppressor cells (MDSCs) in inflammatory and autoimmune diseases, cancer, and in response to infectious agents.^6–8^ MDSCs are a heterogeneous population of immature cells that can impair immune responses. They can suppress the responses of T cells, natural killer (NK), and antigen-presenting cells through suppressive mechanisms such as arginase, nitric oxide (NO), reactive oxygen species (ROS), programmed death ligand-1 (PD-L1), and indoleamine 2,3-dioxygenase 1 (IDO-1).^8,9^ Although a few studies have addressed the involvement of MDSCs in fungal infections, the role of these cells in PCM was recently investigated.^6,8^ The first study by Preite et al.^6^ observed that MDSCs are present and have a negative impact on the severity of the disease. Transferring these cells to mice infected with *P. brasiliensis* resulted in a higher fungal load, greater lung injury, evidenced by Colony Forming Units (CFU) counts and histopathology, and reduced survival rates. Furthermore, depletion of MDSCs using anti-Gr1 led to an increase in the immune response of Th1 and Th17 cells, which may have contributed to disease control.

Considering the limitations of current treatments for PCM, 5-Fluorouracil (5-FU) is an interesting candidate for the treatment of this systemic mycosis. It is a chemotherapeutic drug first reported in the early 1950s and later approved by the US Food and Drug Administration (FDA) for the treatment of various types of cancer.^10^ Ghiringhelli and Apetoh^11^ highlight one of the main characteristics of 5-FU: its ability to stimulate the immune response. In mice bearing thymic tumors, the authors demonstrated that 5-FU selectively eliminated circulating MDSCs through apoptosis without affecting B lymphocytes, T lymphocytes, or NK cells.^12^ Another study showed that 5-FU selectively targets cells with low expression of thymidylate synthase, which is a characteristic of tumor cells and MDSCs, but not other immunological cells.^13^ In fact, MDSCs have even lower thymidylate synthase expression than tumor cells, making the low dose of 5-FU more specific to MDSCs and keeping other leukocytes safe.^12,13^

In addition, another study conducted by Preite et al.^14^ demonstrated that administration of 5-FU to *P. brasiliensis*-infected mice promoted a partial, but specific depletion of MDSCs. Consequently, there was an improvement in disease control, alongside diminished fungal burden in target organs, increased survival rates, and enhanced protective antifungal responses, such as Th1 and Th17. Importantly, a significant reduction in MDSC frequencies was observed in animals treated with 5-FU at 4, 6, and 8 weeks of infection. Moreover, the protection induced by 5-FU was reversed when MDSCs were transferred back into the animals.^14^ These results indicate that the removal of MDSCs is a key factor responsible for the immunological improvement observed in 5-FU-treated mice. Taken together, the objective of this study is to evaluate the efficacy of combined 5-FU and AmB therapy in a murine model of chronic PCM, as well as its impact on immune response against this important fungal pathogen.

## Results

The primary objective of our study was to identify the most suitable antifungal agent to combine with 5-FU. To this end, three antifungal agents were evaluated for their efficacy in reducing fungal burden, as measured by colony-forming units (CFU) in mice that were intratracheally infected with *P. brasiliensis* yeasts. The first agent tested, amphotericin B (AmB), administered at 5 mg/kg, resulted in a reduction in the fungal burden across all examined organs, including the lungs, liver, and spleen (Figure 1A–C). The second agent, itraconazole, induced a reduction in CFU only in the spleen, and solely at the highest dose of 20 mg/kg (Figure 1D–F). Finally, fluconazole reduced fungal burden in only one organ—the liver—at the maximum concentration of 20 mg/kg (Figure 1G–I). These results indicate that AmB was the most effective antifungal in this initial screening, achieving significant reductions in all three organs at a dose of 5 mg/kg. The inclusion of AmB is particularly relevant given its widespread use in cases of systemic dissemination (Carolus et al.). Considering that the focus of this study is the chronic phase of PCM, during which the infection has already disseminated to multiple organs and T cells become exhausted (Loures et al.; Galdino et al.), this choice is highly appropriate for the experimental model.

**Figure 1:**
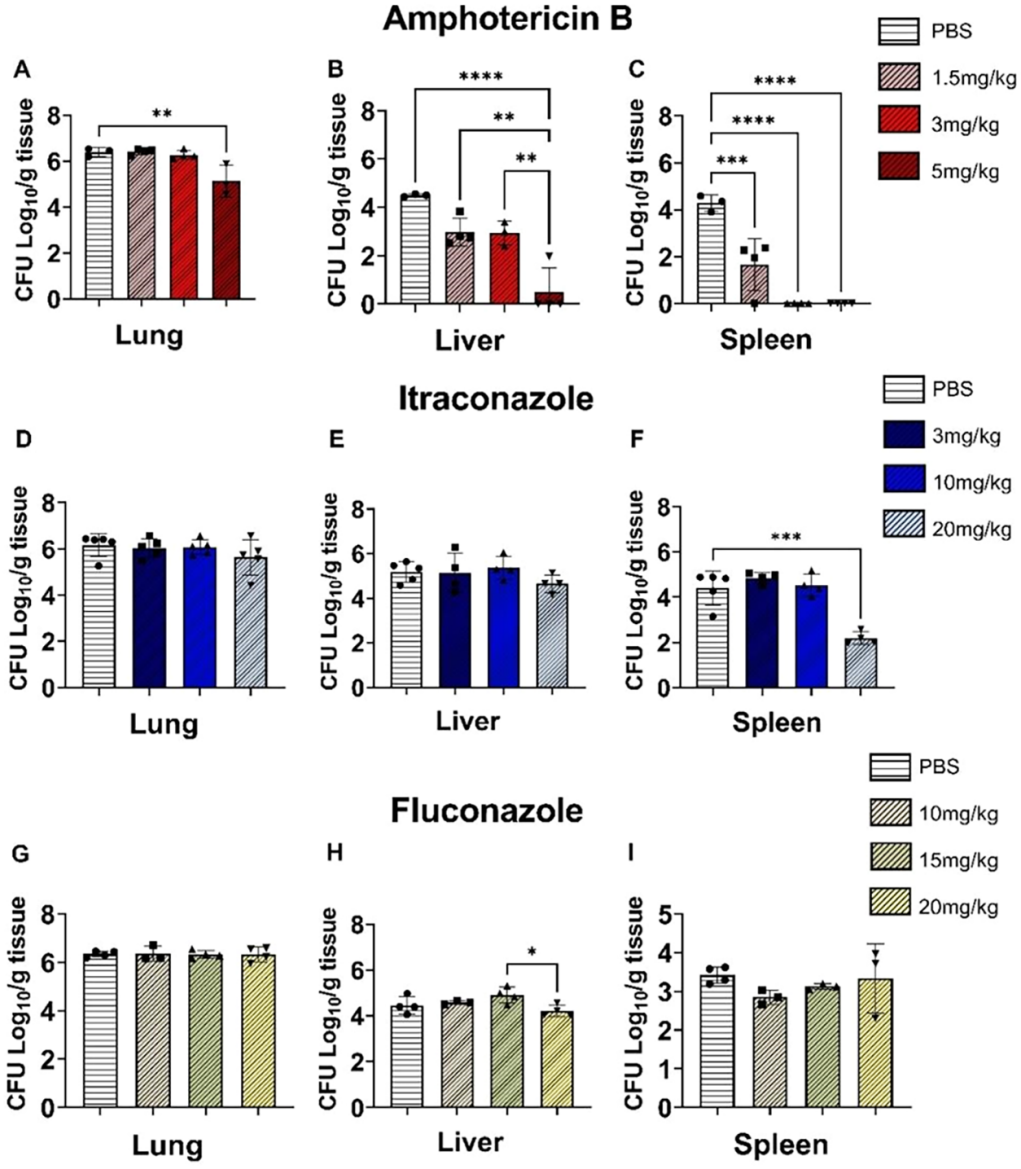
Effect of different doses of AmB (A–C), itraconazole (D–F), and fluconazole (G–I) on the fungal burden in the lungs, liver, and spleen. C57BL/6 mice were infected intratracheally with 1 × 10^6^ *P. brasiliensis* yeasts. After 6 weeks of infection, groups of mice were intraperitoneally treated with three different doses of each antifungal agent on alternate days for 2 weeks. PBS was used as control. CFU counts in lungs, liver, and spleen were determined after 8 weeks of infection. The data presented represent three experiments conducted with 3–5 mice/group. Comparisons between groups and means ± standard deviation were analyzed by ANOVA. Bars represent mean ± SD, based on four mice per sample. Values were considered significant when *p < 0.05, **p < 0.01, ***p < 0.001 and ****p < 0.0001.

Having chosen the best antifungal agent, we proceeded to evaluate the combined therapy of 5-FU+AmB for 2 weeks of treatment (Figure 2A). The animals receiving the combined 5-FU+AmB treatment exhibited a significant reduction in the fungal burden in the lungs and spleen compared with those treated with either agent alone. This indicates enhanced therapeutic efficacy and improved control of the infection, as reduced fungal load reflects a more effective host response and decreased pathogen persistence (Figure 2B–D). Additionally, a reduction in CFU counts was observed in the lungs, liver, and spleens of animals treated individually with 5-FU and AmB compared to the control/PBS group. Similar results were observed in the assessment of lung damage. Animals receiving individual treatments showed reduced organ injury compared to the PBS group, while those treated with 5-FU+AmB therapy exhibited significantly less pulmonary damage compared with animals treated with 5-FU or AmB as single therapy (Figure 2F). It is worth noting that the lungs of animals in the 5-FU+AmB group exhibited a healthier appearance, with a greater reduction in granulomas (regions formed by clusters of *P. brasiliensis* and immune cells) compared to all other groups (Figure 2H).

**Figure 2:**
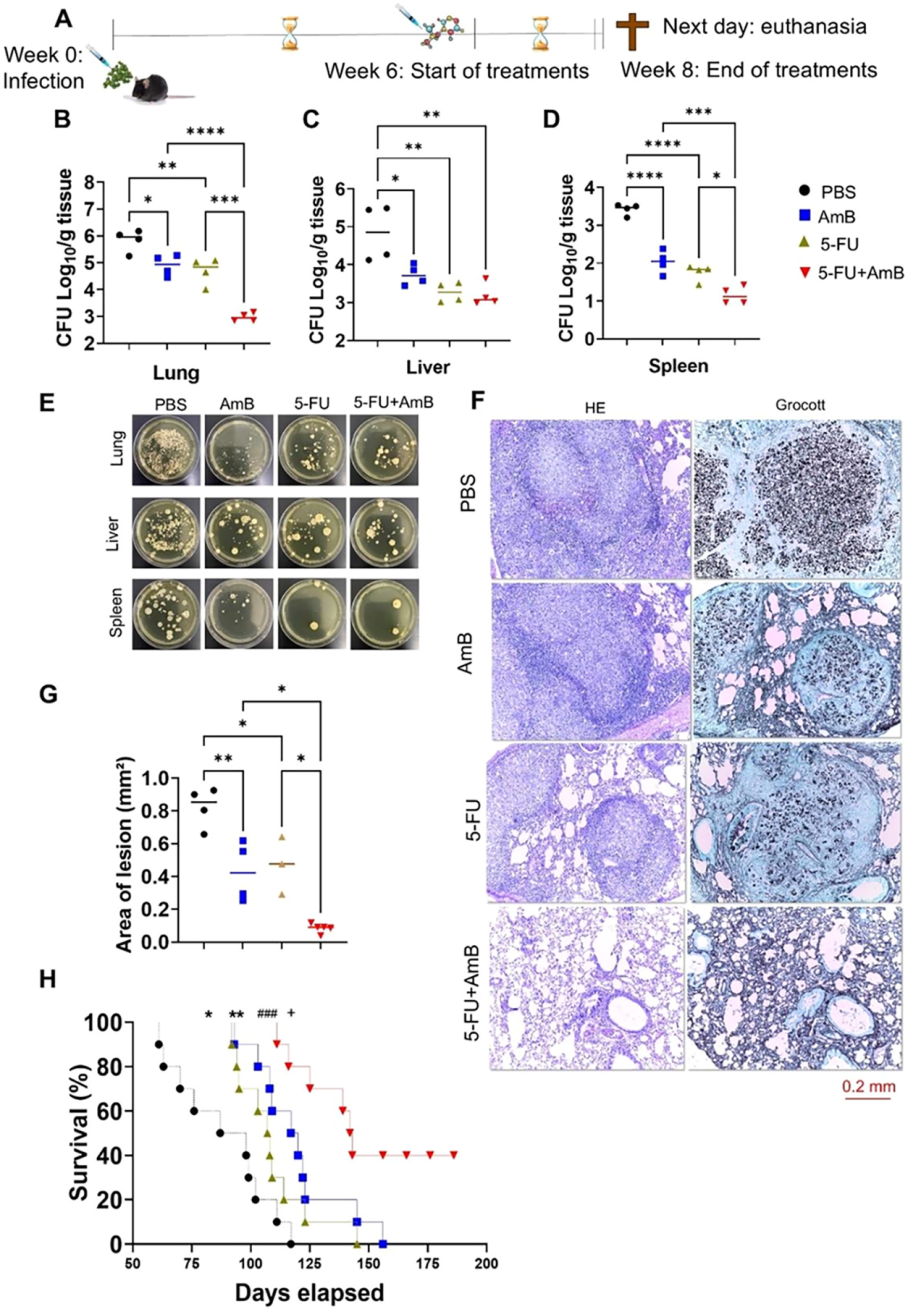
Effect of 5-FU in combination with amphotericin B on murine Paracoccidioidomycosis. (A) C57BL/6 mice were infected intratracheally with 1 × 10^6^ *P. brasiliensis* yeasts. After 6 weeks of infection, mice were treated via the intraperitoneal route with 5 mg/kg AmB and 20 mg/kg 5-FU on alternate days for 2 weeks. Control group received PBS. CFU counts in (B) lungs, (C) liver, and (D) spleen were assessed after 8 weeks of infection. The data represents three experiments, each conducted with 4–5 mice. Comparisons between groups and means ± standard deviation were analyzed by ANOVA. Bars represent mean ± SD. Values were considered significant when: *p < 0.05; **p < 0.01; ***p < 0.001 and ****p < 0.0001. (E) The images representing the results were taken on the final day of colony counting (15 days after plating the cell suspension), using samples from the lungs at a 1:100 dilution, the liver at a 1:10 dilution, and undiluted spleen samples. (F) Histopathology was performed with hematoxylin-eosin and Grocott staining—10× magnification. (G) Analysis of the pulmonary tissue. The total area of lung lesions was calculated in square micrometers of 5 microscopic fields per slide. Comparisons between two groups, mean ± SD, were analyzed by ANOVA. Values were considered significant when: *p < 0.05; **p < 0.01 and ****p < 0.0001. (H) The survival curve analysis was performed in a single experiment with *n* = 10 animals/group and used the Log-rank test (Mantel-Cox) to compare the two groups. Values were considered significant when: *p < 0.05 and **p < 0.01 compared to the PBS group; +*p* < 0.05 compared to the AmB group and ###*p* < 0.001 compared to the 5-FU group.

All these improvements contributed to increased survival in mice receiving the combined therapy. Initially, animals treated with either 5-FU or AmB alone showed improved survival compared with the PBS group. Survival in the PBS group started to decline after 60 days of infection, whereas animals treated with individual agents maintained higher survival rates up to approximately 100 days. Notably, in the 5-FU+AmB group, survival only declined around day 125 postinfection and remained at 40% even after the other groups had reached their end point (Figure 2G). These findings demonstrate a significant improvement in the survival of mice treated with the combined therapy compared with all other groups.

As noted, the combined treatment (AmB+5-FU) proved to be more effective than the individual therapies. Following this, we evaluated the physiological impact of the treatments on the animals. During the development of new therapies, evaluating renal and hepatic biomarkers is essential for ensuring safety and understanding pharmacokinetics and toxicity profiles. Monitoring these markers allows early detection of organ toxicity, helps in dose selection, informs safety margins, enables understanding of drug-induced liver injury (such as that caused by the antifungal AmB) or nephrotoxicity, and ensures that potential adverse effects are identified before human trials or during later stages.^15,16^ A reduction in body weight was observed in animals treated with PBS, AmB and 5-FU+AmB after 2 weeks of treatment (at the time of euthanasia), compared to their initial body weight at the start of treatment (Figure 3A). Regarding renal function, a significant increase in serum creatinine was observed exclusively in animals treated with AmB, suggesting that this drug may induce mild nephrotoxic effects (Figure 3C). Conversely, alterations in hepatic function were detected only in the combined 5-FU+AmB group, evidenced by increased ALT levels compared with the control and 5-FU groups, indicating a potential synergistic impact of the combined therapy on hepatic metabolism or hepatocellular integrity (Figure 3D).

**Figure 3:**
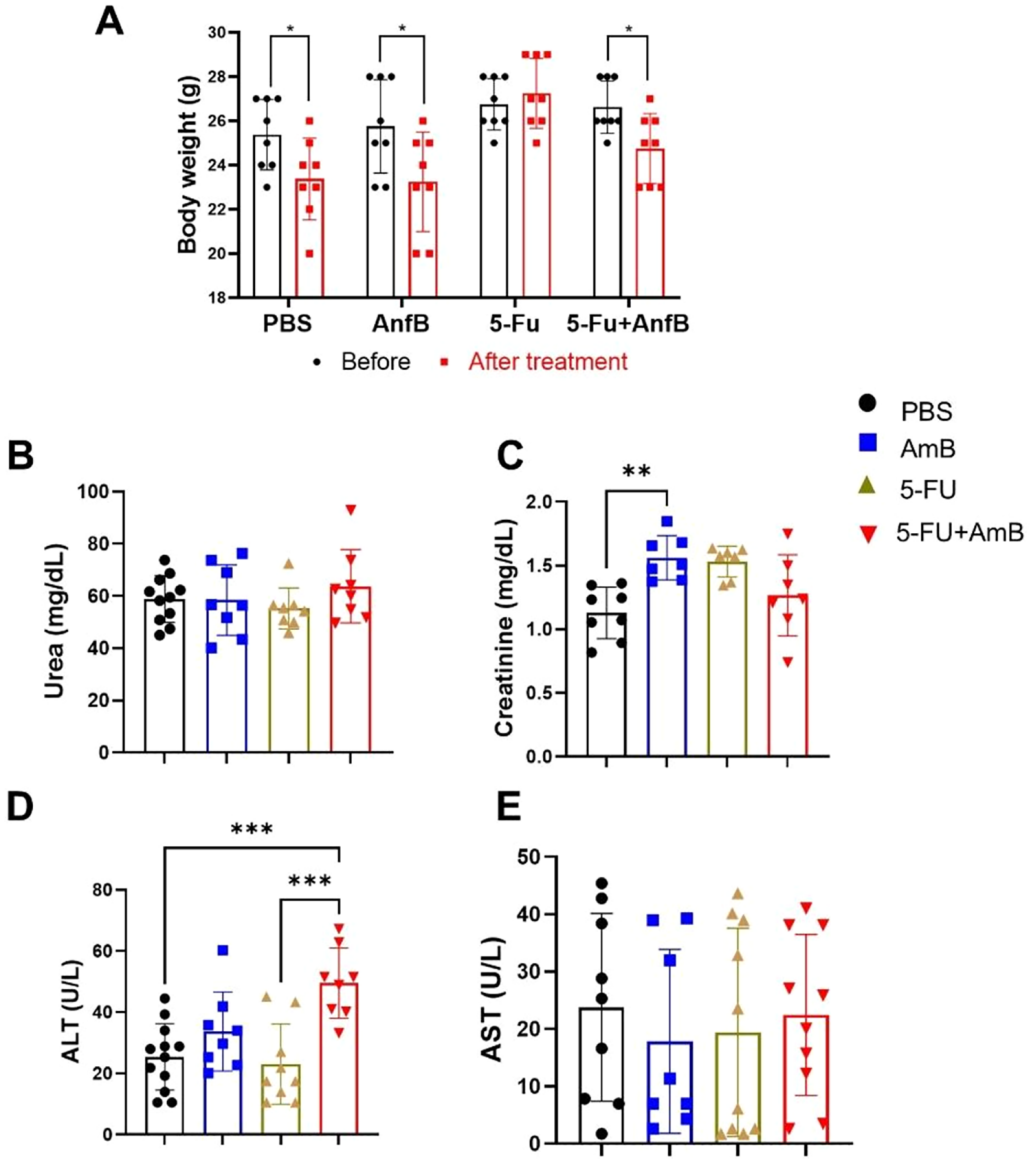
Evaluation of biochemicals markers. (A) Determination of body weight. C57BL/6 mice were infected through the intratracheal route with 1 × 10^6^ *P. brasiliensis* yeasts. After 6 weeks of infection, the animals were treated intraperitoneal with a dose of 5 mg/kg AmB and 20 mg/kg 5-FU on alternate days for 2 weeks. Control animals received PBS. Body weight (grams) was assessed at the beginning and end of treatment (between the sixth and eighth week). Comparisons between initial and final weights were analyzed using Student’s *t* test. Bars represent mean ± SD, based on eight mice per group. Values were considered significant when *p < 0.05. (B–C) Analysis of renal and (D, E) hepatic function markers. Comparisons between groups, means ± standard deviation obtained were analyzed by ANOVA. Bars represent mean ± SD, based on 8 to 12 animals/group. Values were considered significant when: **p < 0.01 and ***p < 0.001.

Monitoring immune response dynamics is essential when exploring therapeutic strategies that involve suppressor cell depletion across various diseases, including cancer and chronic infections. Excessive or prolonged depletion of MDSCs could result in uncontrolled inflammation, as these cells are critical for limiting tissue damage caused by immune overactivation. Furthermore, elucidating the immune modulation induced by the combined 5-FU and AmB therapy provides new insights into its therapeutic potential, as 5-FU depletes MDSCs, thereby relieving immunosuppression and strengthening the host immune response against infection.^14^ The impact of treatments on antifungal responses was assessed by two approaches: quantification of cytokines in the lung, liver and spleen; and analysis of infiltrating leukocytes in the lung. Regarding the pro-inflammatory cytokines IL-1β and TNF-α, treatment with AmB resulted in a reduction of IL-1β levels in the lungs and liver compared with the PBS group. In animals treated with the combined therapy with 5-FU+AmB, IL-1β levels increased in the lungs (compared to the AmB group) but decreased in the liver (compared to the 5-FU group). Additionally, the combined therapy led to an increase in TNF-α levels in the liver when compared with the AmB group. In contrast, 5-FU monotherapy reduced TNF-α levels in the lungs compared with the PBS group (Figure 4A–B).

**Figure 4:**
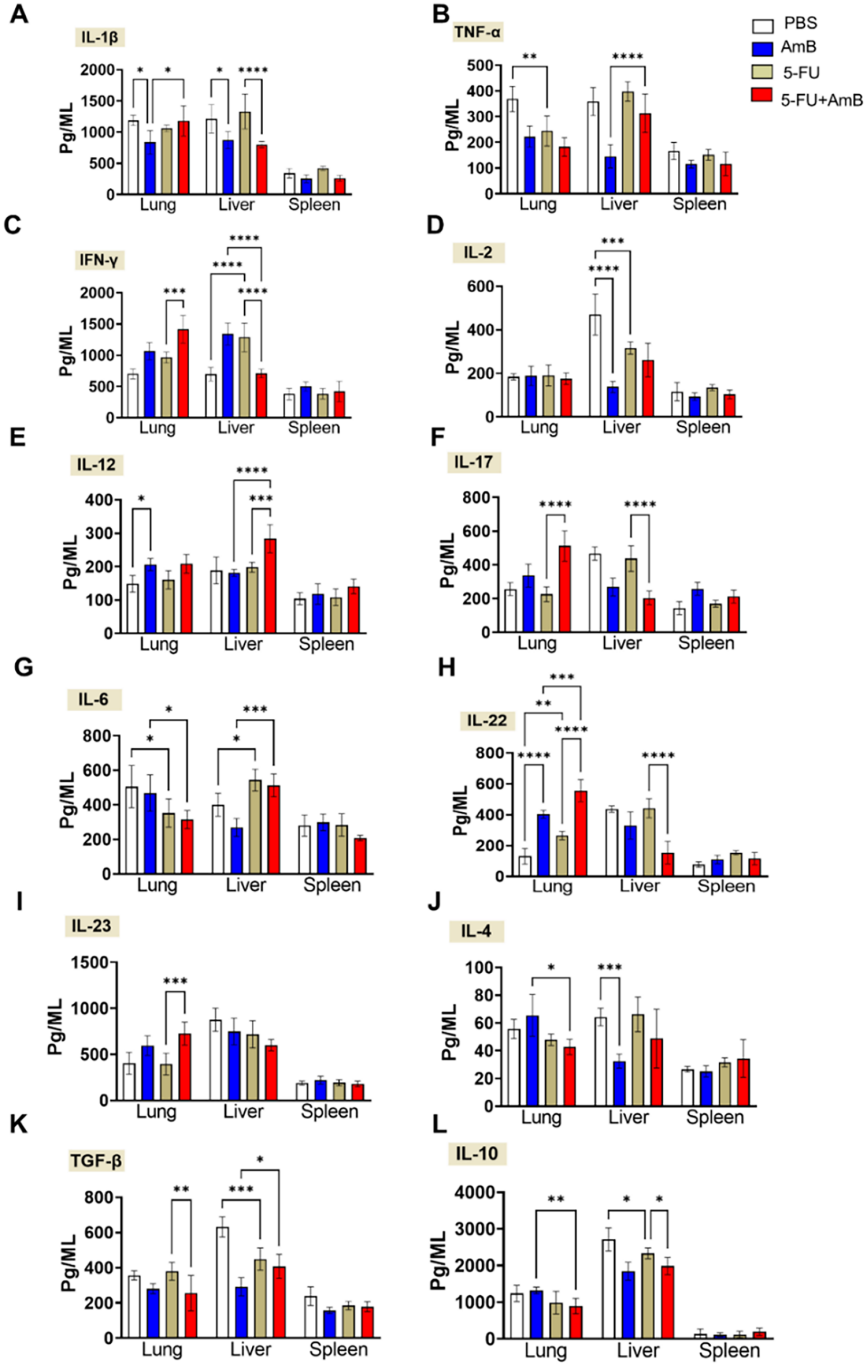
Influence of treatments on cytokine secretion in lungs, liver, and spleen homogenates after 8 weeks of infection. C57BL/6 mice were infected intratracheally with 1 × 10^6^ *P. brasiliensis* yeasts. After 6 weeks of infection, the animals were treated i.p. with 5 mg/kg AmB and/or 20 mg/kg 5-FU on alternate days for 2 weeks. Control animals received PBS. Lung, liver, and spleen were obtained, and cytokines were quantified by ELISA from the organ homogenate. IL-1β (A), TNF-α (B), IFN-γ (C), IL-2 (D), IL-12 (E), IL-17 (F), IL-6 (G), IL-12 (H), IL-23 (I), IL-4 (J), TGF-β (K) and IL-10 (L). The data presented represents three experiments conducted with 4–5 mice each. Comparisons between groups and means ± SD were analyzed by ANOVA. Bars represent means ± standard error, based on four mice per sample. Values were considered significant when: *p < 0.05; **p < 0.01; ***p < 0.001 and ****p < 0001.

Cytokines expressed by Th1 cells, such as IFN-γ, IL-2, and IL-12, are associated with better disease outcome in both humans and murine models of PCM.^17,18^ In the present study, increased levels of IFN-γ were observed in the lungs of animals treated with 5-FU+AmB compared with those treated with 5-FU alone. In the liver, IFN-γ levels were higher in animals receiving 5-FU compared with the PBS group. However, in the 5-FU+AmB group, IFN-γ levels in the liver were reduced compared to both the AmB and 5-FU monotherapy groups (Figure 4C). Regarding IL-2, a reduction was observed in the liver of treated with either 5-FU or AmB compared with the PBS group (Figure 4D). Additionally, IL-12 levels in the lung were increased in animals treated with AmB compared to the PBS group. While in the liver, animals receiving the combined 5-FU+AmB treatment showed higher IL-12 levels compared with those receiving either treatment alone (Figure 4E).

Th17 differentiation is driven primarily by TGF-β and IL-6, whereas IL-23 is required for Th17 maintenance and expansion; these cytokines stimulate IL-17/IL-22 production and neutrophil recruitment, contributing to granuloma formation and host protection during PCM.^19,20^ An increase in IL-17 levels was observed in the lungs of animals treated with 5-FU+AmB compared to those treated with 5-FU alone. In contrast, IL-17 levels in the liver were reduced in the 5-FU+AmB group compared to the 5-FU group (Figure 4F). IL-6 levels were reduced in the lungs of animals treated with 5-FU compared to the PBS group, and in animals receiving 5-FU+AmB compared to those treated with AmB alone. Conversely, IL-6 levels were increased in the liver of animals treated with 5-FU compared to PBS, and in the 5-FU+AmB group compared to the AmB group (Figure 4G). Moreover, an increase in IL-22 levels was observed in the lungs of animals receiving either 5-FU or AmB monotherapy compared to the PBS group, and this increase was even more pronounced in animals treated with the combined 5-FU+AmB therapy (Figure 4H). IL-23 levels were increased only in the lungs of animals treated with 5-FU+AmB compared to those treated with 5-FU alone (Figure 4I).

Patients with disseminated *Paracoccidioides brasiliensis* infection—where the pathogen extends beyond a single site, such as the lungs, to involve multiple organs or systems—exhibit elevated levels of Th2-associated cytokines, including IL-4.^18^ In our study, animals receiving the combined treatment showed reduced levels of IL-4 in the lungs, compared to those treated with AmB alone (Figure 4J). Elevated levels of the immunosuppressive cytokines TGF-β and IL-10 have been associated with the suppression of cellular immune responses in patients with PCM, particularly through the activity of regulatory T cells (Tregs). These cytokines contribute to the inhibition of effector T cell functions, thereby facilitating pathogen persistence and disease progression.^21^ In the lungs, levels of both cytokines were reduced in the group receiving the combined treatment, with TGF-β levels lower than those in the 5-FU group, and IL-10 levels lower than in the AmB group. In the liver, animals treated with 5-FU+AmB showed increased TGF-β levels compared to the AmB group, alongside reduced IL-10 levels compared to the 5-FU group (Figure 4K,L).

To assess whether the treatments affected the frequency and number of leukocytes during *P. brasiliensis* infection, flow cytometry was performed on lung-infiltrating leukocytes from mice (Figure 5A,B). Myeloid cells were characterized using anti-CD45 and anti-CD11b antibodies. No significant changes were observed in the frequency and number of these cells (Figure 5C–D). Within the myeloid cell population, our analysis focused primarily on macrophages (Mφ), dendritic cells (DCs), and neutrophils. Mφ play a role in modulating the immune response in PCM. M1-polarized macrophages, characterized by the expression of TNF-α and MHC-II molecules, contribute to the intracellular elimination of the fungus.^22,23^ In the present study, animals treated with AmB showed a reduction in both the frequency and number of Mφ compared to those treated with PBS. In contrast, animals receiving the combined 5-FU+AmB treatment exhibited an increase in the frequency and number of Mφ compared to those treated with 5-FU alone (Figure 5E,F).

**Figure 5:**
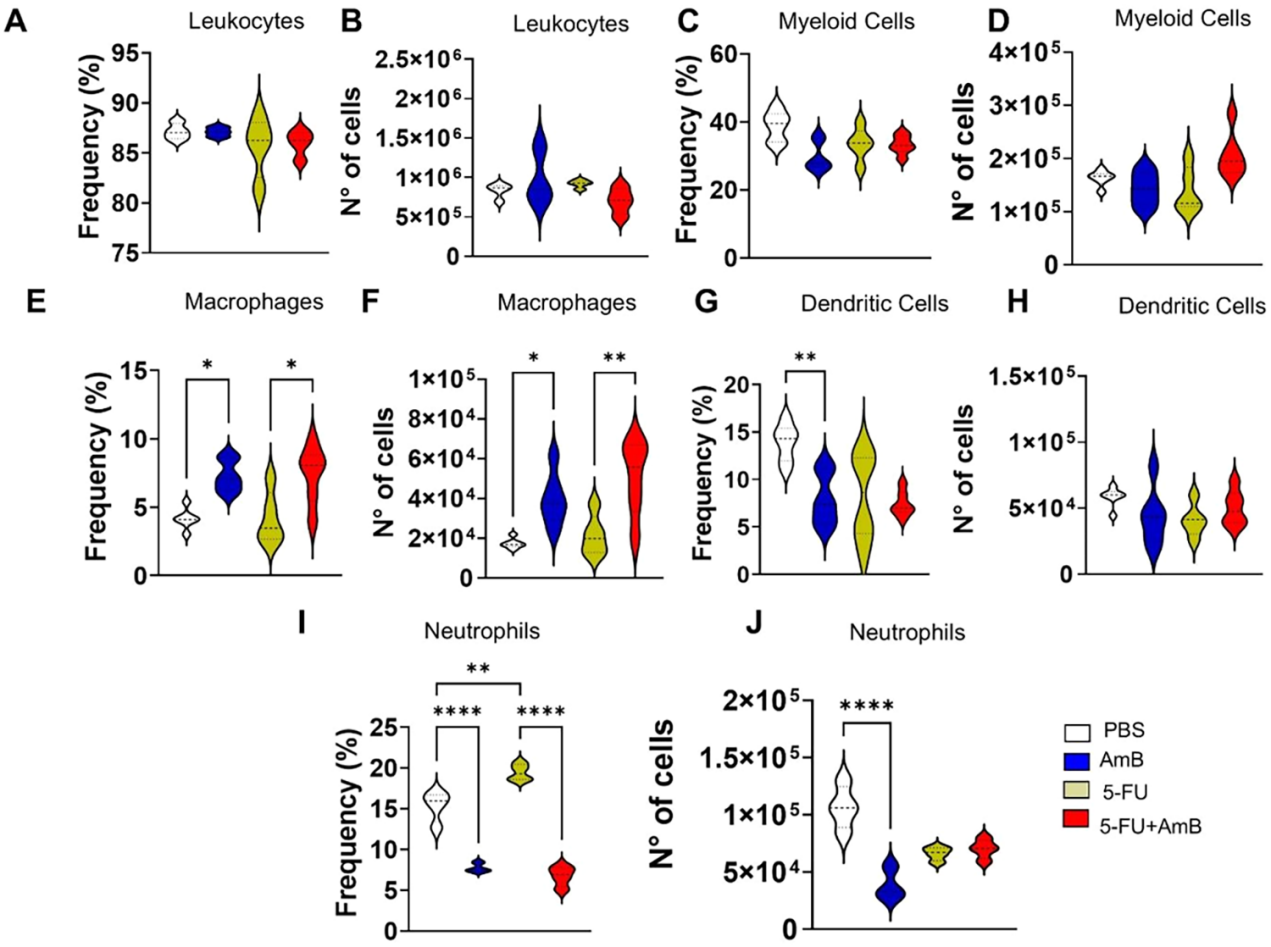
Frequency and number of leucocytes (A, B), myeloid cells (C–D), macrophages (E, F), dendritic cells (G, H) and neutrophils (I, J) in the lung-infiltrating leucocytes. C57BL/6 mice were infected through the intratracheal route with 1 × 10^6^ *P. brasiliensis* yeasts. After 8 weeks of infection, the animals were euthanized and the lungs were collected, macerated and subjected to enzymatic digestion. Dead cells were excluded from the analysis using a Live/Dead antibody and the leukocyte populations were identified using the following phenotypes: CD45^+^ (only leukocytes); CD45^+^CD11b^+^ for myeloid cells; CD45^+^CD11b^+^F4/80^+^ for macrophages; CD45^+^CD11b^+^CD11c^+^F4/80^–^ for dendritic cells; and CD45^+^CD11b^+^Ly6G^hight^ for neutrophils. Data analysis was done by Flowjo software and comparisons between groups, averages ± the standard deviation obtained were analyzed by ANOVA. The data refers to a representative experiment of three. The bars represent averages ± o SD of 4–5 mice/group.

Dendritic cells (DCs) are professional antigen-presenting cells with the unique ability to direct the adaptive immune response during the activation of naive T cells.^24,25^ In this regard, a reduction in DCs frequency was observed only in the lungs of animals treated with AmB compared to those treated with PBS (Figure 5G,H). Neutrophils are associated with protection and immune regulation during the early phase of the disease. However, they have been shown to be detrimental in the chronic stage, as they are associated with fibrosis.^26^ Following treatments, a reduction in both the frequency and number of neutrophils was observed in animals treated with AmB alone, compared to those receiving PBS. In contrast, treatment with 5-FU increased neutrophil frequency compared to PBS. Finally, the combined 5-FU+AmB therapy resulted in a reduction in neutrophil frequency compared to 5-FU monotherapy (Figure 5I,J).

Surface markers such as CD40, CD80 and CD86 on macrophages and dendritic cells are critical for initiating and regulating adaptive immune responses. These molecules provide the necessary signals for T cell activation.^27^ An increase in both the frequency and number of CD40^+^Mφ was observed in animals treated with 5-FU+AmB compared to those treated with 5-FU alone (Figure 6A,B). The combined treatment also reduced both the frequency and number of macrophages expressing MHC class II compared to animals treated with 5-FU alone. Furthermore, AmB monotherapy reduced the levels of CD40^+^DC compared to the PBS group, whereas the 5-FU+AmB combination increased the frequency of these cells compared to AmB monotherapy (Figure 6I). Finally, the frequency of CD86^+^DC was diminished in animals treated with either AmB or 5-FU+AmB compared to those receiving PBS (Figure 6M).

**Figure 6:**
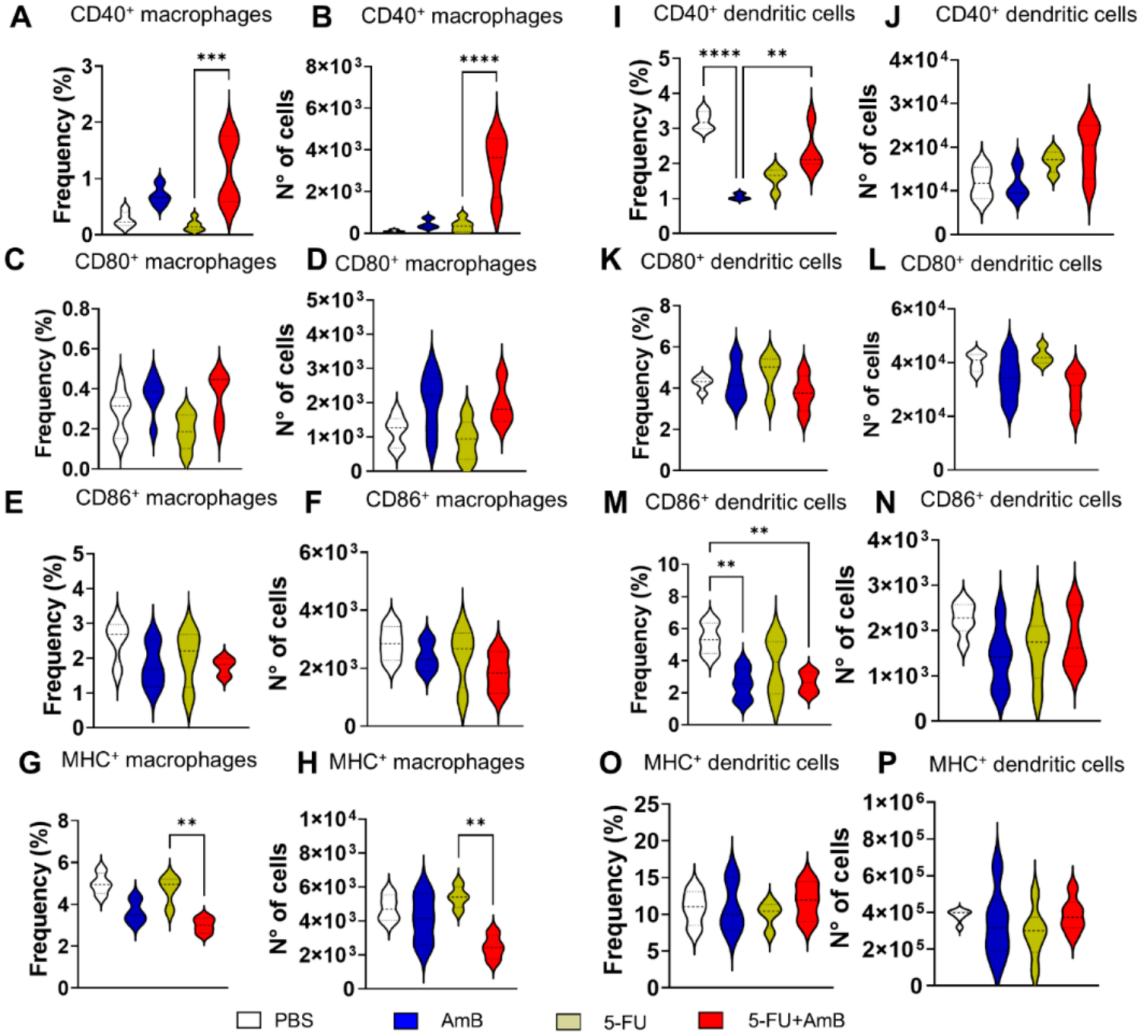
Frequencies and numbers of macrophage and dendritic cells. C57BL/6 mice were infected through the intratracheal route with 1 × 10^6^ *P. brasiliensis* yeasts. After 8 weeks of infection, the animals were euthanized, and the lungs were collected, macerated, and subjected to enzymatic digestion to obtain infiltrating leukocytes. Macrophages expressing activation molecules (CD40, CD80, CD86 and MHC-II) were defined as CD45^+^CD11b^+^CD11c^+^F4/80^+^ cells (A-H). Dendritic cells expressing the same activation molecules were identified as CD45^+^CD11b^+^CD11c^+^F4/80^–^ cells (I-P). The data refers to a representative experiment of three. The bars represent means ± standard error of 4–5 mice/group and the values were considered significant when **p < 0.01, ***p < 0.001 and ***p < 0.0001.

We also used flow cytometry to evaluate the influence of MDSCs depletion (through 5-FU treatment) combined with AmB on T lymphocytes. Animals treated with 5-FU alone showed a reduction in the frequency of CD4^+^ lymphocytes compared to the PBS group, whereas the combined 5-FU+AmB treatment increased the number of these cells compared to the 5-FU group (Figure 7A,B). CD4 lymphocytes activation was assessed by analyzing the CD25 marker. Interestingly, the combined treatment reduced both the frequency and number of activated CD4^+^CD25^+^ lymphocytes compared to animals treated with 5-FU alone (Figure 7C–D).

**Figure 7:**
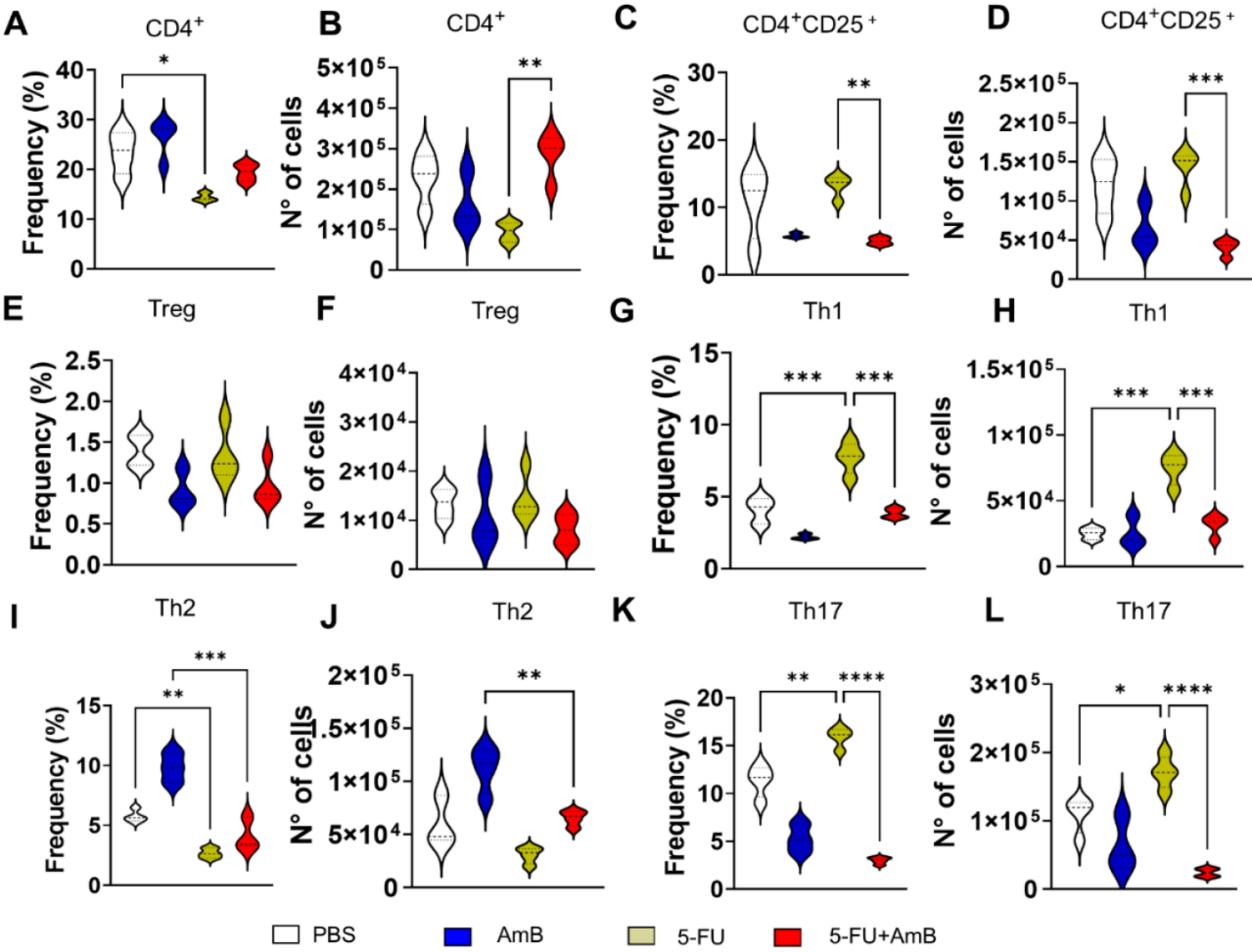
Frequency and number of CD4 T lymphocytes. C57BL/6 mice were infected through the intratracheal route with 1 × 10^6^ *P. brasiliensis* yeasts. After 8 weeks of infection and treatment, the animals were euthanized and the lungs were collected, macerated, and subjected to enzymatic digestion to obtain infiltrating leukocytes. Lymphocytes populations were identified as CD4^+^ (A-B) and CD4^+^CD25^+^ (C-D) cells. Intracellular staining was performed to identify Tregs as CD4^+^CD25^+^Foxp3^+^ (E-F); Th1 (CD4^+^IFN-γ^+^(G-H); Th2 as (CD4^+^IL-4^+^(I-J); Th17 as (CD4^+^IL-17^+^(K-L); and Tregs (CD4^+^CD25^+^Foxp3^+^). Data analysis was performed using FlowJo software, and comparisons between groups, means ± standard deviation obtained, were analyzed by ANOVA. The data refers to a representative experiment of three. Bars represent mean ± SD of 4–5 mice per group.

T helper and Treg responses were also analyzed. An increase in both the frequency and number of Th1 cells was observed in animals treated with 5-FU compared to the PBS group. However, the combined 5-FU+AmB treatment resulted in a reduction in these parameters compared to 5-FU alone (Figure 7G,H). Regarding the Th2 population, 5-FU treatment resulted in decreased levels compared to PBS, while the combined therapy reduced further reduced both the frequency and number of Th2 cells compared to the AmB group (Figure 7I,J). Similarly, the frequency and number of Th17 cells were elevated in the 5-FU group relative to PBS-treated animals but were significantly reduced in animals receiving the combined treatment compared to those treated with 5-FU alone (Figure 7K,L).

Tregs play a significant role in modulating the immune response during *P. brasiliensis* infection. These cells help maintain immune tolerance and prevent excessive inflammation by suppressing the activation and proliferation of effector T cells. In the context of PCM, Tregs can influence disease progression by modulating the balance between protective immunity and immunopathology.^28,29^ In animals that received the combined treatment, Treg levels remained unchanged (Figure 7E,F), suggesting that the response of these animals is balance between immune defense and the prevention of excessive immunosuppression.

An increase in CD8^+^T cells frequency was observed in animals treated with AmB compared to the PBS group (Figure 8A). The frequency and number of activated CD8^+^T cells, identified by CD69 expression, were elevated in 5-FU-treated animals compared to the PBS group (Figure 8C–D). However, the combined 5-FU+AmB therapy led to a reduction in both the frequency and number of these activated cells compared to 5-FU alone. Similarly, the frequency and number of Tc1 cells were increased in animals treated with 5-FU relative to PBS, while a decrease was observed in the combined treatment group compared to 5-FU monotherapy (Figure 8E,F). Regarding Tc17 cells, an increase in frequency was detected only in animals treated with AmB alone compared to the PBS group (Figure 8I). Overall, the combined treatment led to a reduction in several lymphocyte subpopulations compared to individual therapies, including activated T CD4 cells, as well as Th1, Th2, and Th17 subsets. Additionally, it suppressed the activation of CD8^+^ T cells and the TC1 population.

**Figure 8:**
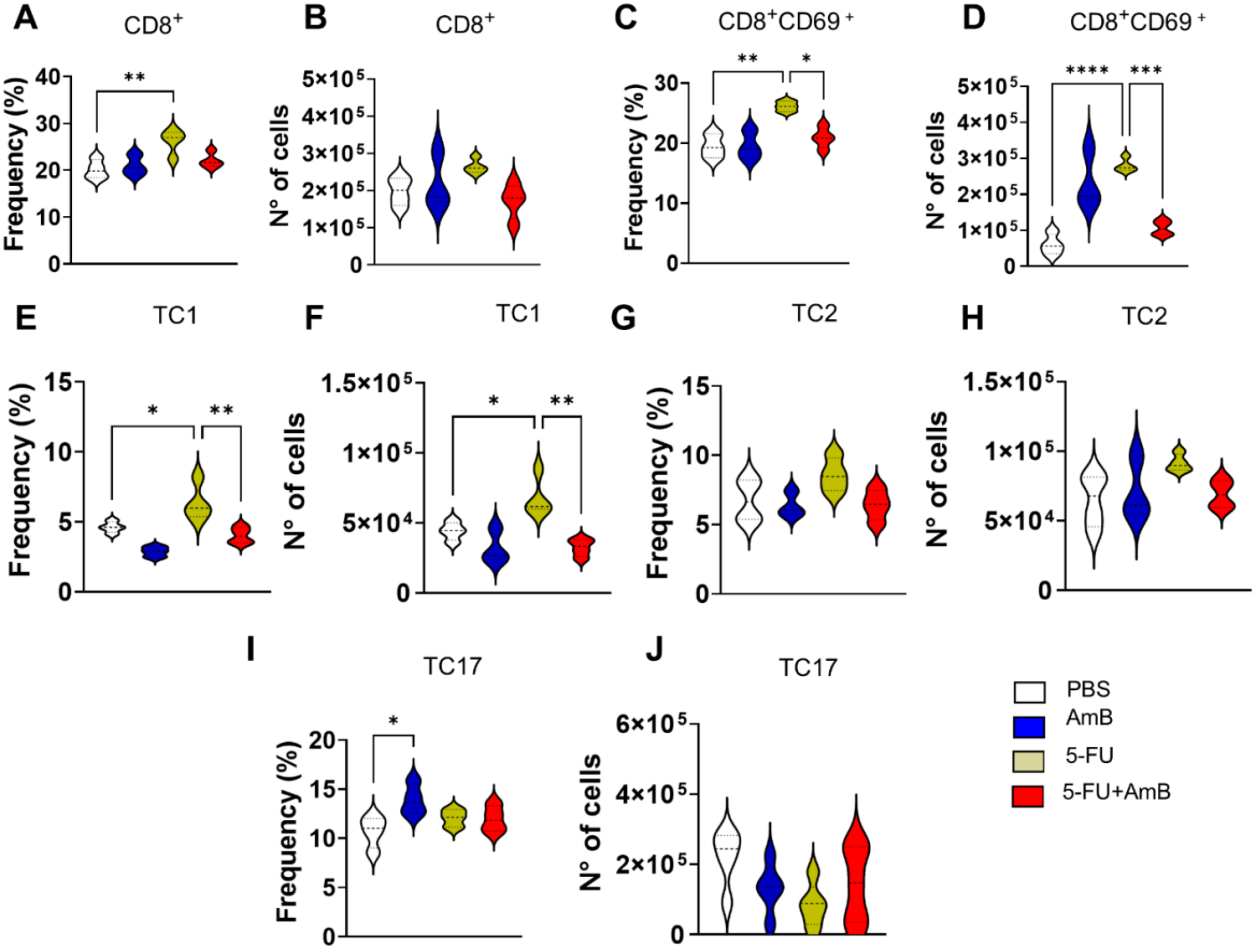
Frequency and number of CD8 T lymphocytes. C57BL/6 mice were infected through the intratracheal route with 1 × 10^6^ *P. brasiliensis* yeasts. After 8 weeks of infection, the animals were euthanized and the lungs were collected, macerated, and subjected to enzymatic digestion to obtain infiltrating leukocytes. CD8 lymphocytes populations were identified as CD8^+^ (A-B) and CD8^+^CD69^+^ cells (C-D). Intracellular staining was performed to identify Tc1 (CD4^+^IFN-γ^+^(E-F); Tc2 as (CD4^+^IL-4^+^ (G-H); and Tc17 (CD4^+^IL-17^+^ (I-J). Data analysis was performed using FlowJo software, and comparisons between groups, means ± standard deviation obtained, were analyzed by ANOVA. The data refers to a representative experiment of three. Bars represent mean ± SD of 4–5 mice per group.

Although our results demonstrated a reversal in disease progression, they were insufficient to completely eradicate the fungal burden. Therefore, we considered whether extending the duration of therapy could enhance pathogen clearance. Based on this, we evaluated another experimental protocol: the multiple treatment (MT). This protocol consisted of a 6-week infection period followed by three 2-week treatment cycles, each separated by a 1-week interval (Figure 9A). Figure 9B shows that multicycle treatment (MT) with 5-FU+AmB significantly reduced the fungal burden in the lungs compared to treatment with 5-FU alone. Moreover, the mean CFU (Log10/g tissue) in the combined group was approximately 1.5, which represents a 50% reduction compared to the mean value observed in the 8-week protocol (CFU Log10/g tissue = 3.0, see Figure 2). Among the five mice in this group, no fungal colony growth was observed in two, suggesting possible eradication of the fungus. These findings are consistent with the analysis of lung lesion areas (Figure 9C–D), which also showed a reduction in pulmonary lesions in animals treated with 5-FU+AmB compared to those receiving monotherapies. When compared to the previous protocol, lesion regression was observed not only in the 5-FU+AmB group but also in animals treated with AmB alone. Furthermore, the survival analysis in animals receiving the new treatment showed a decline only after 150 days postinfection, with most individuals remaining alive beyond 200 days, while the animals in the group that received only one cycle of treatment had already reached the experimental end point (Figure 9E). Notably, in the single-cycle treatment group, mortality occurred between days 100 and 150, consistent with the survival data shown in Figure 1G. Of the six animals that survived the assay, the lungs of three are shown in Figure 9F. Larger granulomas can be seen in only one of them, while another has smaller granulomas (middle panel), and the first animal has most of its pulmonary alveoli preserved. These results indicate that the proposed protocol holds therapeutic potential for PCM in a murine model.

**Figure 9:**
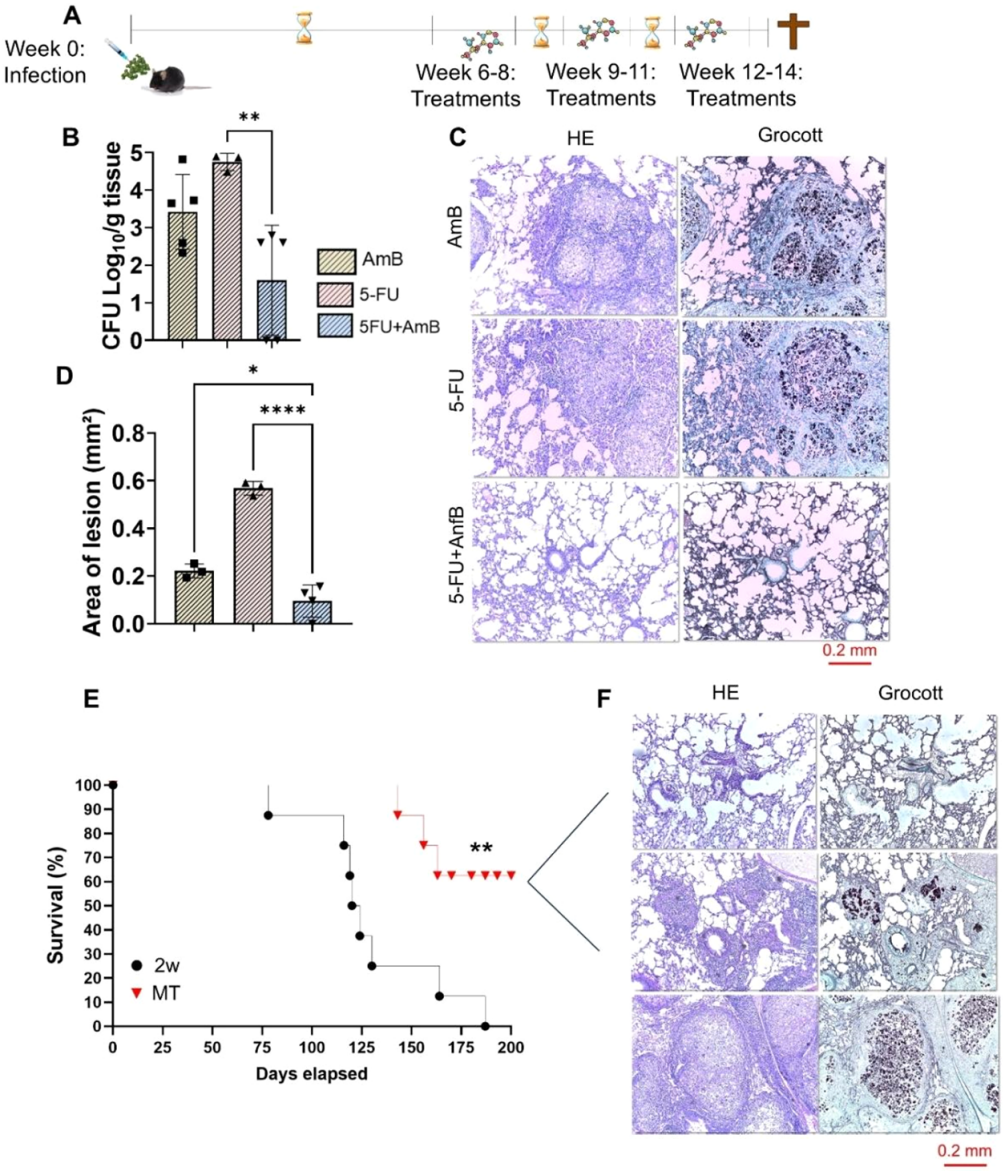
Effect of treatment with 5-FU in combination with amphotericin B in a 14-week protocol. (A) C57BL/6 mice were infected through the intratracheal route with 1 × 10^6^ *P. brasiliensis* yeasts. After 6 weeks of infection, the animals were treated i.p. with 5 mg/kg AmB and 20 mg/kg 5-FU every other day for 2 weeks for 3 cycles with an interval of 1 week each. Euthanasia was performed after 14 weeks of infection. (B) CFU counts in lungs. (C) Representative histological images of the lungs of animals in the AmB, 5-FU, and 5-FU+Amb groups that received multiple treatments in the 14-week protocol. (D) Analysis of the lesion area of the lungs of the three groups. (D) Histopathology was performed with hematoxylin-eosin and Grocott—10× magnification. The data presented represent two experiments conducted with 4–5 mice each. Comparisons between groups and means ± standard deviation were analyzed by ANOVA. Bars represent mean ± SD (E) The survival curve analysis was performed in a single experiment with *n* = 8 animals/group and used the Log-rank test (Mantel-Cox) to compare the two groups. (F) Representative histological images of the lungs of three of the last six animals that received multiple treatments. Values were considered significant when: *p < 0.05, **p < 0.01 and ****p < 0.0001.

In summary, the 5-FU+AmB combination effectively reduces fungal burden, prolongs survival, and attenuates the inflammatory response after only 2 weeks of treatment, demonstrating significant therapeutic potential. Moreover, additional treatment cycles may further enhance fungal clearance. These findings support the consideration of extending the treatment protocol to achieve complete disease eradication.

## Discussion

Our research group has primarily focused on identifying effective molecules for the treatment of PCM, with particular emphasis on the modulation of the immune response—such as the depletion of MDSCs. These cells have been reported as key players in the pathogenesis of several chronic infections.^30,31^ However, our group was the first to report evidence of the detrimental role of this cell population in pulmonary PCM, and depleting this population using 5-FU resulted in a better disease outcome.^6,8,14,32^

Few studies have investigated the potential therapeutic benefits of combining 5-FU with other agents, whether antifungals or otherwise, in fungal infections. Król et al.^33^ evaluated the effects of combined therapy with 5-FU and folinic acid against *C. albicans*. The combination was effective against 15 fluconazole-resistant *Candida* strains, whereas 5-FU alone did not produce significant reductions in these strains. Nevertheless, it is worth noting that although folinic acid exhibits antifungal properties, its use is more commonly associated with cancer treatment, as is the case with 5-FU.

Based on these findings, we aimed to evaluate a combination therapy using 5-FU with some of the most used antifungal agents for the treatment of this disease. The literature shows the recurrent use of AmB, itraconazole, and fluconazole in various fungal infections.^34,35^ In our first experiments, AmB (5 mg/kg) reduced the fungal load in the lung, liver, and spleen of animals infected with *P. brasiliensis*, even resulting in the eradication of the fungus in the spleen. Considering its effectiveness along with the fact that this drug has been recommended since 1958 for the treatment of the most severe cases of PCM,^36^ we chose this antifungal for combination with 5-FU.

In fact, groups receiving the combined therapy exhibited a significant reduction in fungal burden in the lungs and spleen compared to the groups treated with AmB or 5-FU alone. This reduction, indicating an attenuation of disease severity, was also reflected in lung histology by decreased tissue lesions. Importantly, increased survival was observed in the combined treatment group relative to those receiving individual therapies.

Because MDSCs are suppressive cells, depleting them can alter the cytokine and immune cell profiling. Thus, we measured cytokine secretion by ELISA and evaluated changes in cell population by flow cytometry. Our results showed an increase of type-1 and type-17 cytokines for the combined therapy group, with higher secretion of IL-1β, IFN-γ, IL-22, IL-17, and IL-23 compared with mice treated with AmB or 5-FU alone. Th1/Th7 have been associated with a better disease outcome in PCM, since they provide a protective effect.^17,19^ In addition to the cytokines, we observed an increase in the frequency of macrophages and a decrease of neutrophil in the lungs of AmB + 5-FU treated mice compared with 5-FU treatment alone. M1-polarized macrophages are fundamental in PCM, since they are related to the intracellular killing of *P. brasiliensis*.^22^ This increase in macrophages corroborates with the type-1 cytokine profiling and explains the lower CFU for this group. Furthermore, the decrease of neutrophils frequency also contributes to the better disease outcome for the 5-FU+AmB mice, since it has been reported that depleting neutrophils during chronic PCM promotes the resolution of pulmonary inflammation and fibrosis.^37^

Additionally, we observed a decreased titer of some anti-inflammatory cytokines, such as IL-4, IL-10, and TGF-β, in the lungs of mice treated with combined therapy when compared to monotherapy. Those cytokines have been reported to have a direct detrimental role in chronic PCM, since they are related to a harmful Th2 profile.^38–40^ Notably, a lower frequency of Th2 was observed in the 5-FU+AmB group. Another cytokine that was found in lower levels is IL-6 that plays a crucial role in regulating the balance between Th17 cells and Treg cells. IL-6, along with TGF-β, induces the development of Th17 cells from naive T cells. In contrast, IL-6 inhibits TGF-β-induced Treg differentiation.^41,42^ The reduced presence of TGF-β and IL-6 could explain the lower frequency of Th17 cells in the 5-FU+AmB group compared with single-therapy controls. Moreover, the reduced Th17 could explain the decrease in neutrophil as well.

Regarding antigen presentation, we observed a reduced frequency of DCs expressing the costimulatory molecule CD86 and lower frequency of macrophages expressing the antigen-presenting molecule MHC-II. These results might indicate an impaired antigen presentation by those cells.^43^ However, despite this potential impairment, the disease remained controlled, as evidenced by the lower fungal burden, reduced lung lesion areas, and increased survival. Interestingly, both macrophages and DCs showed increased expression of CD40 when compared to the mono therapy groups, which could indicate that those cells were more active.^44^

The combined therapy not only displayed alterations in the innate immunity, but also in the adaptive immunity. While the number of CD4^+^ T cells increased compared with 5-FU alone, these cells exhibited reduced activation, as evidenced by lower CD25 expression. Although no changes in Treg frequency were observed, treatment with 5-FU+AmB reduced both the frequency and number of Th1 and Th17 cells compared with 5-FU monotherapy. Even though a Th1/Th17 displays a protective effect during chronic PCM, the combined treatment was indeed better than the monotherapy in controlling the infection.

A partial downmodulation of the Th1 response pattern leads to mild or moderate chronic forms of human disease and resistance in murine models of PCM.^17^ In contrast, a shift toward a Th2 response pattern results in acute/subacute, chronic, and severe forms in patients, as well as susceptibility in mice. Th1 and Th2 responses were characterized by total or partial containment of the fungus in compact granulomas and fungal dissemination of disorganized granulomas, respectively. Moreover, Arruda et al.^45^ demonstrated that susceptibility to *P. brasiliensis* infection cannot be attributed to an intrinsic inability to develop cellular immune response. A single immunization procedure can result in opposite disease outcomes. This study demonstrates that immunoprotection can be achieved through a balanced immune response. Our findings expand on this concept by revealing a scenario in which effective pathogen clearance occurs alongside a broad downmodulation of T-helper responses. Although this pattern may initially appear paradoxical, it reflects a favorable trajectory of the immune response rather than an impairment. The decline in Th1, Th2, and Th17 cells indicates a reduction in inflammation that is proportionate to the decreasing fungal burden, as directly supported by our CFU data. This coordinated decrease, observed alongside histopathological resolution and enhanced survival, indicates a transition from aggressive, pathogen-driven inflammation toward a stabilized equilibrium. Thus, the combined 5-FU and AmB treatment appears to promote a return to immune homeostasis by effectively eliminating the infectious stimulus, thereby reducing the need for a sustained, high-magnitude inflammatory response.

Moreover, the treatment of infected mice with the combined therapy also altered CD8^+^ cells when compared to the treatment promoted by 5-FU alone. Whereas a decrease in activation—evidenced by reduced CD69—was observed. Additionally, a lower frequency and number of Tc1 was seen when comparing those two groups. In murine PCM, an enhanced Tc1 response has been correlated with protective immunity against Paracoccidioides infection,^46^ further supporting our findings.

It is worth noting that the additional protocol consisting of three treatment cycles showed promising results compared to the two-week treatment protocol, with complete fungal clearance observed in the lungs of 2 out of 5 animals in the combination therapy group. Survival rates in this group were also improved. However, the data obtained thus far suggest that two-weeks treatment period is already sufficient to demonstrate significant efficacy as a new experimental therapy for PCM. Previous studies have shown that the optimal duration of antifungal therapy depends on the type and severity of the infection and must also be tailored to the immune status and therapeutic response of the host—whether animal or human. While some fungal infections require several months or even years, prolonged antifungal use may lead to more severe side effects and increase the risk of antifungal resistance, a growing concern in clinical settings.^47–49^ The use of 5-FU in combination with AmB may help mitigate this issue, as 5-FU is a chemotherapeutic agent with a mechanism of action distinct from conventional antifungals and may pose a lower risk of inducing antifungal resistance.

A known limiting factor in the clinical use of AmB is its potential to induce nephrotoxicity.^4^ This antifungal is predominantly distributed to lipid-rich tissues, exhibits low penetration into the central nervous system, and has high plasma protein binding, all of which contribute to its long terminal half-life and tissue accumulation. Its pharmacokinetics are also influenced by formulation: liposomal preparations reduce renal toxicity and alter the volume of distribution.^50^ Two widely used biomarkers of kidney damage are blood urea nitrogen and creatinine.^51^ In our analysis, animals treated with AmB alone showed a reduction in serum creatinine levels, which does not correspond to the expected pattern of toxicity but has been previously reported. For example, Medoff et al.^52^ reported alterations in creatinine, insulin, and urea levels indicative of impaired renal function following clinical treatment with AmB.

Additionally, 5-FU exhibits rapid systemic distribution after administration, readily crosses cell membranes, and undergoes extensive hepatic metabolism via the enzyme dihydropyrimidine dehydrogenase (DPD), which accounts for its rapid elimination and short half-life. Small variations in DPD activity can significantly affect the drug’s toxicity and efficacy.^53^ The combined treatment resulted in increased ALT levels compared to both the PBS group and the group treated with 5-FU alone. This effect may be related to AmB’s known interaction with hepatic cytochrome P450 enzymes, which can influence the liver’s metabolic capacity.^54^ However, the incidence of severe acute or subacute hepatotoxicity caused by AmB remains low. Current evidence indicates that AmB is not metabolized in the liver, with renal elimination being its primary route.^55^ Therefore, exploring combined therapy with 5-FU and AmB in chronic PCM patients may represent a promising strategy to improve life expectancy.

## Conclusion

Two weeks of combination therapy with 5-fluorouracil and amphotericin B resulted in improved disease outcomes in a murine model of PCM. This was evidenced by reduced fungal load, decreased pulmonary tissue damage, and increased survival of animals infected with *P. brasiliensis*. In addition, increasing the number of treatment cycles enhanced efficacy, as shown by lower fungal loads and higher survival rates compared to animals receiving a single cycle. Modulation of the immune response - specifically through the reduction of lymphocyte activation and the Th1, Th2, and Th17 responses in lung tissue—further supports these findings, as a balanced immune profile is associated with improved infection control. Our data indicate that restoring immune function through MDSCs depletion with 5-FU, in combination with AmB, leads to more effective control of PCM than either treatment alone. This study highlights the potential of 5-FU as part of a combination therapy strategy in PCM, aimed at reducing treatment duration and minimizing the adverse effects associated with prolonged antifungal therapy.

## Methods

### Animals

Male wild-type C57BL/6 mice, aged 8 to 12 weeks, were used. Initially sourced from Jackson Laboratories, these animals were acquired as specific pathogen-free from the Center for the Development of Experimental Models for Biology and Medicine (CEDEMEUNIFESP) and subsequently maintained in the NB2 vivarium of the UNIFESP Institute of Science and Technology. All procedures involving mice were approved by the UNIFESP Animal Ethics Committee (No. 3128270224).

### Infection with *P. brasiliensis*

The virulent Pb18 strain of the fungus *P. brasiliensis*, maintained by weekly subcultures in Fava Netto medium at 37 °C, was used. The fungus was collected and washed with phosphate-buffered saline (PBS, pH 7.2). Mice were anesthetized and subjected to intratracheal (i.t.) infection as previously described.^56^ Briefly, after intraperitoneal (i.p.) injection of ketamine 90 mg/kg and xylazine 10 mg/kg, the animals were infected with 1 × 10^6^ yeasts contained in 50 μL of PBS by i.t. inoculation, which allows direct distribution of fungal cells to the lungs.

### Combined Treatment of 5-FU with AmB

Six weeks after *P. brasiliensis* infection, groups of four mice received injections equivalent to 20 mg/kg of the chemotherapy agent.^14^ AmB was chosen as the antifungal agent because it is the most widely used drug in the treatment of the chronic form of PCM and because it presented the best results in a pilot trial in our laboratory (Figure 1). After 6 weeks of infection, three treatment protocols were performed: (i) treatment maintained for 2 weeks every 48 h with euthanasia the following day after 8 weeks of infection and (ii) three cycles of treatments maintained for 2 weeks every 48 h, with 1-week intervals; euthanasia the following day after 14 weeks of infection.

### CFU Assays, Survival Rates, and Histological Analysis

The number of viable yeasts in the infected organs (lung, liver, and spleen) was determined by counting the number of Colony Forming Units (CFU), as established by Singer-Vermes et al.^57^ Survival studies were performed with groups of 10 mice. Deaths were recorded daily. For histological examinations, the left lung and liver of infected mice were removed and fixed in 10% formalin. Five-micrometer sections were stained with hematoxylin-eosin (for lesion analysis) and silver (for fungal evaluation). Morphometric analysis was performed using a Nikon DXM 1200c camera and Nikon NIS AR 2.30 software.

### Body Weight Assessment and Biochemical Analysis

To assess the impact of antifungal treatment combined with 5-Fu, the animals’ body weight was monitored before and after treatment. Furthermore, liver function markers alanine aminotransferase (ALT) and aspartate aminotransferase (AST) were measured, as well as renal function markers creatinine and urea nitrogen using Labtest colorimetric diagnostic kits. After euthanasia, blood was collected in syringes containing EDTA. The samples were centrifuged at 1500 rpm for 10 min at 4 °C. Approximately 10 μL of serum was used to measure each biomarker, according to the kit package insert. The mice were not fasted.

### Cytokines Detection

The lungs, livers, and spleens of infected animals were removed and individually dissociated in 5 mL of PBS. The supernatants were separated from cellular debris by centrifugation at 3000*g* for 10 min and stored at −80 °C. Cytokine levels were measured by ELISA immunoassay, according to the manufacturers’ recommendations, as follows: IL-1β (Cat. No 887013–Invitrogen), IL-2 (Cat. No 431001–Biolegend), IL-4 (Cat. No 431101–Biolegend), IL-6 (Cat. No 431301–Biolegend), IL-10 (Cat. No 887105-Invitrogen), IL-12 (Cat. No 433604–Biolegend), IL-17 (Cat. No 432501-Biolegend), IL-22 (Cat. No 887422–Invitrogen), IL-23 (Cat. No 887230–Invitrogen), TNF-α (Cat. No 430901–Biolegend), IFN-γ (Cat. No 887314–Invitrogen), and TGF-β (Cat. No BMS6084–Invitrogen). Plates were read using a spectrophotometric plate reader (VersaMax, Molecular Devices).

### Flow Cytometry for Characterization of Lung Infiltrating Leucocytes

Myeloid cell and lymphocyte subpopulations were measured by multiparametric flow cytometry. Cells from the lungs of infected animals were stained for total leukocytes (CD45^+^), total myeloid (CD45^+^CD11b^+^), dendritic cells (CD45^+^CD11b^+^CD11c^+^F4/80^–^), alveolar macrophages (CD45^+^CD11b^+^F4/80^+^), and neutrophils (CD45^+^CD11b^+^Ly6G^higt^) to determine whether the treatments would interfere with other myeloid cell populations. Additionally, dendritic cells and macrophages were stained for CD40^+^, CD80^+^, CD86^+^ and MHCII^+^. We also analyzed the presence of CD4^+^ and CD8^+^ T lymphocytes, as well as activation markers, CD25^+^ and CD69^+^, respectively. The cell concentration of the obtained cell suspensions was adjusted, and the cells were added to U-bottom plates at 1 × 10^6^ cells/well. The cells were then resuspended in PBS-azide containing 2% fetal bovine serum (FBS). The plates were centrifuged, and the supernatant was discarded. The marker antibodies were added after blocking receptors for the Fc portion of IgG with anti-CD16/32 monoclonal antibodies, with the final volume adjusted to 50 μL/well at the appropriate titter.

For the lymphocyte population, intracellular labeling of IFN-γ (Th1 and Tc1), IL-4 (Th2 and Tc2), IL-17 (Th17 and Tc17) was characterized in CD4^+^ T and CD8^+^ T lymphocytes by flow cytometry. The presence of the transcription factor Foxp3 (Treg) was also analyzed in CD4^+^CD25^+^ T cells. For this purpose, after recovery of the infected lungs, the samples were resuspended at 1 × 10^6^/mL and activated with PMA (50 ng/mL), ionomycin (500 ng/mL) and brefeldin-A (1/1000). After 6 h of stimulation and blockade of cytokine release, cells were extracellularly labeled with anti-CD4, CD8, CD69, and anti-CD25. After permeabilization (Cytofix/Cytoperm Plus, BD Biosciences), they were intracellularly labeled with anti-IFN-γ, IL-4, IL-17, and FoxP3, following the permeabilization kit specifications. After incubation, the plates were washed twice with PBS-azide-FBS, the supernatant was discarded, and the cells were resuspended in a final volume of 200 μL in 2% paraformaldehyde and transferred to tubes for cytometry. All samples were kept on ice and protected from light. A minimum of 50,000 events were acquired on a FACSLyric flow cytometer (BD Biosciences) using FACSuite software (BD Biosciences). The frequency of cell surface markers was analyzed with FlowJo software (Tree Star). The gating strategy adopted is shown in Figure S1.

### Statistical Analysis

The data were initially subjected to an exploratory statistical analysis, assessing the normal distribution of each variable and the homogeneity of variances using the *F* test, considering *p* > 0.05 as normal variables. Parametric analyses (Student’s *t* test, one-way or two-way ANOVA) were used for variables with normal distribution and homogeneity of variance. The Bonferroni test was used as a *post hoc* test. For variables that presented *p* > 0.05 in the *F* test, the nonparametric Mann–Whitney test was used. All results are presented as mean ± standard deviation (SD). Statistical analysis and graphing were performed using GraphPad Prism 9.0 software. For survival curve analysis, the Log-rank (Mantel-Cox) test was used to compare the groups.

## Supplementary Material



## Notes

Gating strategy used in flow cytometry experiments: Selection of viable cells, leukocytes and myeloid cells; identification of macrophages, dendritic cells and neutrophils; identification of CD4^+^ and CD8^+^ T lymphocytes and their subpopulations of interest (PDF)

## Abbreviations

5-FC: 5-fluorocytosine
5-FU: 5-fluorouracil
5-FUR: 5-fluorouridine
ALT: alanine aminotransferase
AmB: amphotericin-B
APCs: antigen presenting cells
AST: aspartate aminotransferase
CFU: colony forming unit
CLG: clorgyline
DCs: dendritic cells
DPD: dihydropyrimidine dehydrogenase
FDA: Food and Drug Administration
G-CSF: granulocyte colony-stimulating factor
GM-CSF: granulocyte-macrophage CSF
i.p.: intraperitoneal
i.t.: intratracheal
IS: immune system
MDSCs: myeloid derived suppressor cells
Mφ: macrophages
PCM: paracoccidioidomycosis
