# Supplementary material for: Synergistic Effect of 5‑Fluorouracil and Amphotericin B in Murine Paracoccidioidomycosis: Immune Modulation and Enhanced Fungal Clearance
Source: ACS Infect Dis. 2025 Dec 31;12(2):750–65. doi: 10.1021/acsinfecdis.5c00944 (PMC12910580; doi:10.1021/acsinfecdis.5c00944)
Supplement: Supplementary file 1 [file id5c00944_si_001.pdf]

SUPPORTING INFORMATION

Synergistic Effect of 5-Fluorouracil and Amphotericin B in Murine Paracoccidioidomycosis: Immune Modulation and Enhanced Fungal Clearance

*Filipe N. Franco[1], Ana Claudia S. dos Santos[1], Bianca V. dos Santos[1], Nycolas W. Preite[1], Coral Molist-Homs[1], Luiz Fernando F. de Oliveira[1], Bruno M. Borges[1,2] and Flavio V. Loures[1]**

[1]Institute of Science and Technology, Federal University of São Paulo, São José dos Campos, Brazil. *E-mail: loures@unifesp.br

[2]Department of Biomedical Sciences and Pathobiology, Virginia Tech University, Blacksburg, VA 24060, USA

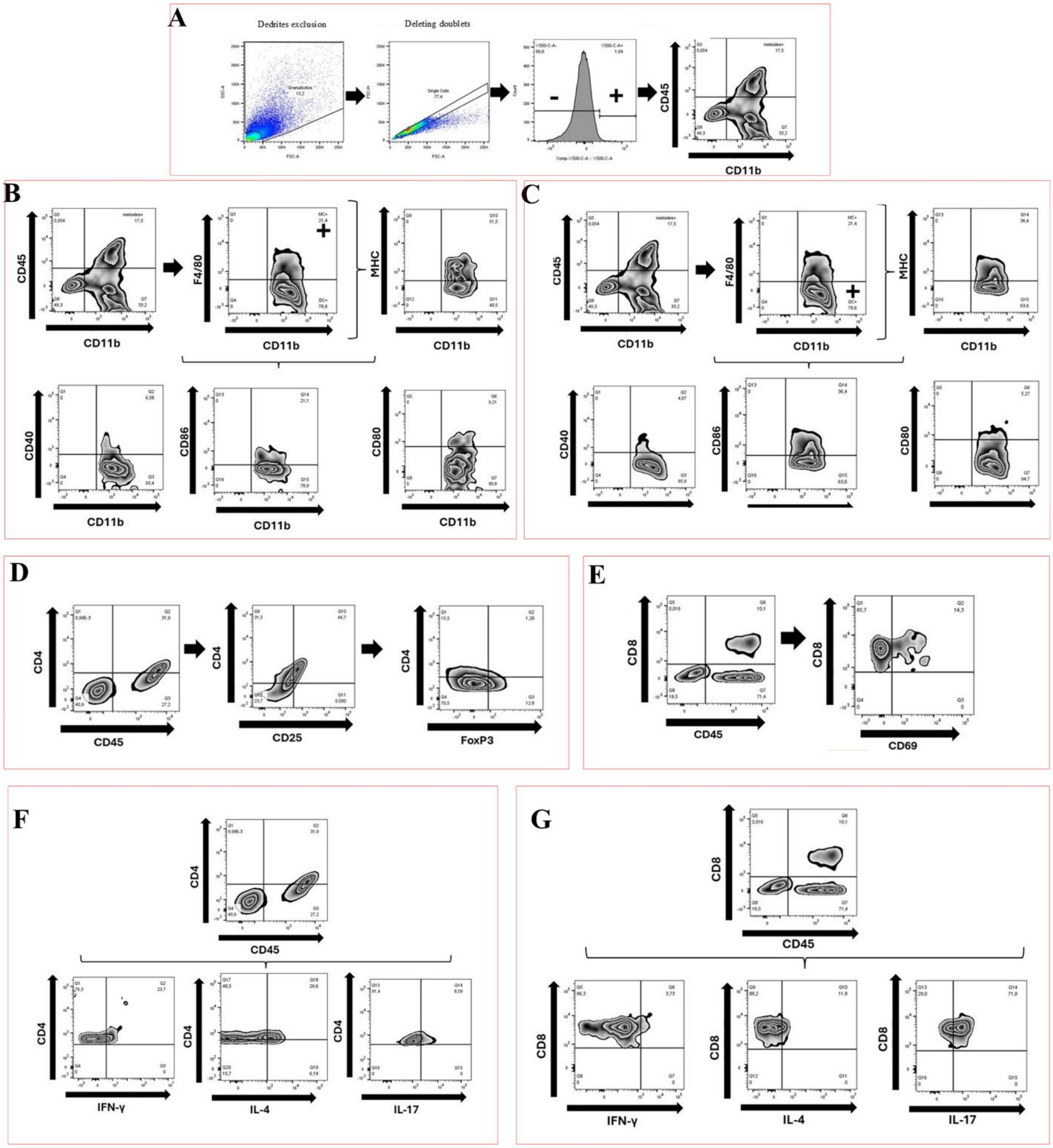

Figure S1. Gating strategy in flow cytometry experiments. Strategy equally adopted in all samples for selection of (A) viable cells, leukocytes and myeloid cells; (B) macrophages; (C) dendritic cells; (D-F) CD4 T and (E-G) CD8 T lymphocytes, as well as subpopulations of interest.
